# Large intraoral spindle cell lipoma

**DOI:** 10.4317/jced.58405

**Published:** 2021-08-01

**Authors:** Iolanda-Zanotelli Lemos, Laís-Romeiro-Lopes-Guerra Cabral, Nícolas-Souza de Souza, Paulo-José-D`Albuquerque Medeiros, Fábio-Ramoa Pires

**Affiliations:** 1 Resident, Oral and Maxillofacial Surgery, Pedro Ernesto University Hospital, Rio de Janeiro State University, Rio de Janeiro/RJ, Brazil; 2 Professor, Oral and Maxillofacial Surgery, Pedro Ernesto University Hospital, Rio de Janeiro State University, Rio de Janeiro/RJ, Brazil; 3 Professor, Oral Pathology, Dental School, Rio de Janeiro State University, Rio de Janeiro/RJ, Brazil

## Abstract

Lipomas are relatively common benign neoplasms composed by mature fat cells. Apart from conventional lipomas, several other subtypes have been described in the oral cavity, including fibrolipoma, myxoid lipoma, angiolipoma, myolipoma, chondrolipoma, osteolipoma and spindle cell lipoma (SCL). Intraoral SCL is rare, representing from 1.4% to 9.8% of all intraoral lipomas. The aim of the present study is to report a case of a large intraoral SCL of the buccal mucosa affecting a 46-year-old male, calling attention to its clinical and histological features and to its successfull surgical conservative management.

** Key words:**Lipoma, spindle cell, oral, buccal mucosa.

## Introduction

Lipomas are relatively common benign neoplasms composed by mature fat cells. Apart from conventional lipomas, several other subtypes have been described in the oral cavity, including fibrolipoma, myxoid lipoma, angiolipoma, myolipoma, chondrolipoma, osteolipoma and spindle cell lipoma (SCL) (1-6). Intraoral SCL is rare, representing from 1.4% to 9.8% of all intraoral lipomas, and few cases have been reported in the English-language literature (1,2,4-6). Furlong *et al*. (3) reported that SCL represented 44% of their intraoral lipomas, but this high frequency is probably due to a bias as the cases were retrieved from a reference center to the referral of more complex cases to second opinion/revision. As some SCL can present clinical and histological features not common in other benign lipomatous tumors, it is important both to clinicians and pathologists to take in account this entity when dealing with lesions containing fat and fibrous tissue. Therefore, the aim of the present study is to report a case of a large intraoral SCL calling attention to its clinical and histological features and to its successfull surgical conservative treatment.

## Case Report

**Figure 1:**
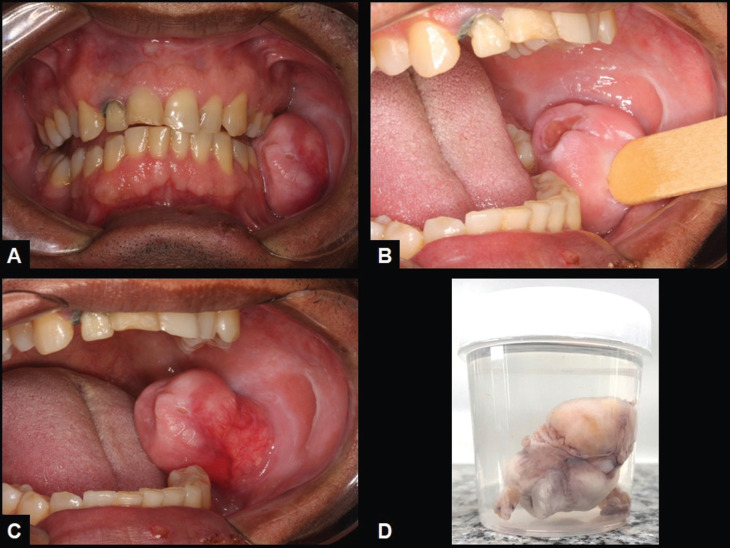
Spindle cell lipoma. Pedunculated soft tissue mass located in the buccal mucosa showing a central area of ulceration (A, B and C). The surgical specimen did not float when immersed in 10% formalin (D).

A 46-year-old male was referred to the Oral and Maxillofacial service complaining of a slow-growing swelling in the face lasting five years. Medical history revealed no significant alterations. Oral examination showed a 5.0 cm fibrous to flaccid submucosal pedunculated nodule covered by normal oral mucosa showing a central 1.0 cm area of ulceration in the left buccal mucosa (Fig. 1A-C). Lesion was asymptomatic, except when the adjacent teeth have contact to it during speeching and mastication. Clinical diagnosis included a benign salivary gland tumor and traumatic fibroma. Due to the pedunculated relationship of the lesion with the adjacent normal mucosa an excisional biopsy was performed under local anesthesia. During surgical removal, it was observed that the lesion had a yellowish appearance resembling adipose tissue, but the surgical specimen did not float when immersed in 10% formalin (Fig. 1D).

**Figure 2:**
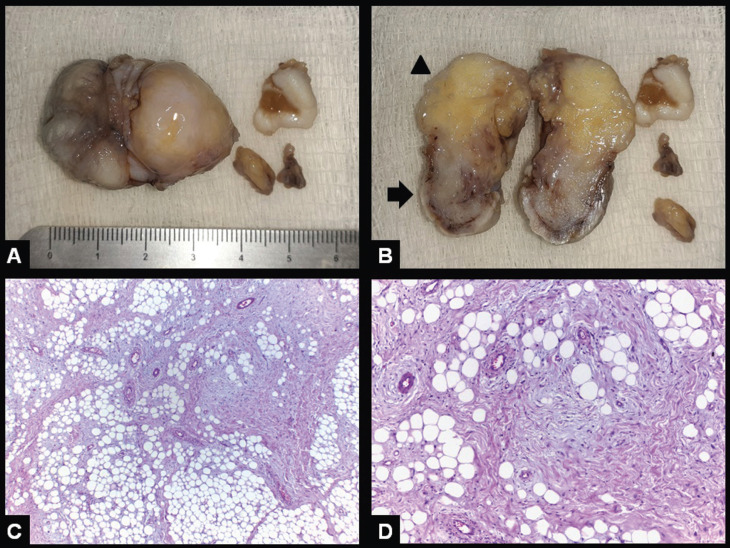
Spindle cell lipoma. Gross examination showing a well-defined soft tissue mass (A) containing whitish fibrous (arrow) and yellowish soft areas (arrowhead) (B). Five µm H&E stained histological sections showing a proliferation of mature adipose cells and spindle cells (C – original magnification 40x; D – original magnification 100 x).

**Figure 3:**
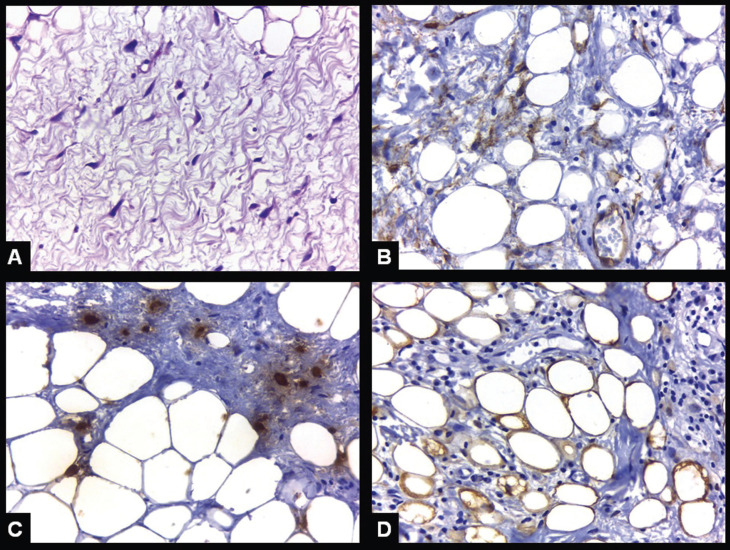
Spindle cell lipoma. Five µm H&E stained histological section showing a detail of the spindle cells with elongated nuclei and bipolar cytoplasm (A – original magnification 400x). Immunohistochemical expression of CD34 in the spindle cells (B), mast cell tryptase in mast cells (C), and S100 protein in mature adipose cells (D) (B, C and D - Immunoperoxidase, original magnification 400x).

The specimen was sent to histological analysis and the gross features revealed that the lesion was partially tan and partially yellowish (Fig. 2A,B). Hematoxylin and eosin stained five µm sections showed the presence of mature adipose cells interspersed by a proliferation of spindle cells with elongated nuclei in a loose fibrous tissue (Figs. 2C,D, 3A). No cellular and nuclear pleomorphism, necrosis or mitotic Figures were observed. Immunohistochemical reactions showed that the spindle cells were CD34 positive, highlighted the presence of numerous mast cells and showed that the mature adipose cells were S100 positive (Fig. 3B-D). Final diagnosis was Spindle cell lipoma. Post operatory period was uneventful and the patient remains in clinical follow-up for nine months with no signs of local recurrence.

## Discussion

SCL was first described by Enzinger and Harvey in 1975 (7), but the first report in the oral cavity was published by McDaniel *et al*. in 1984 (8). Originally, SCL and pleomorphic lipoma, another subtype of lipoma, were described as two separate lesions, but they are now considered to be a spectrum of the same entity (9). Intraoral SCL shows a predilection for males (1,2,10-13) and most affected patients are adults in their fourties to fifties, being rare in childrens and adolescents (1,10-14). SCL manifesting in the oral and maxillofacial region can affect several anatomical locations, including major salivary glands and the oral cavity (3). In the latter, the most common locations are the buccal mucosa, tongue and lips, and less common sites are the floor of the mouth and palate (1-4,10-14).

Most intraoral SCL are relatively small painless lesions measuring less than 4.0 cm, and presenting as a well-circumscribed sessile nodule (1,9-11,13,14). The present case highlights an uncommon clinical presentation for intraoral SCL, as a 5.0 cm pedunculated growth. Another interesting feature in the present case is the presence of ulceration. Clinical differential diagnosis of intraoral SCL is dependent on the anatomical location of the tumor and can include salivary gland tumors, benign mesenchymal neoplasms, mucous extravastion phenomena and mucous cysts (11,13). If one includes a salivary gland tumor and/or a mesenchymal neoplasm in clinical differential diagnosis and the lesion is ulcerated, malignant entities should be also considered. However, other features, such as anatomical limits, consistency, growth rate and insertion in the adjacent normal tissues, should be considered when including a malignancy in differential diagnosis, as ulceration can be secondary to trauma, as shown in the present case. In the present report, although the clinical diagnosis was a salivary gland tumor and the lesion measured 5.0 cm, the surgical procedure performed was an excisional biopsy due to the pedunculated nature of the lesion.

Histologically, oral SCL is characterized by the presence of mature adipose tissue interspersed by a variable amount of spindle cells and mast cells in a background of myxoid and collagenous connective tissue (1,7,9). The spindle cells show elongated nuclei and bipolar cytoplasm (1,7,9). Mitosis and necrotic areas are exceedingly rare, but focal pleomorphic and multinucleated spindle cells can be observed (1,7,9). The spindle cells are positive to CD34 and vimentin, and are negative to most other mesenchymal markers, such as actins and desmin, while the adipose cells are positive to S100 protein. It is important to highlight that some SCL can show a very focal, almost absent, adipose component, and immunohistochemistry is essential as an adjunctive diagnostic tool especially in these cases (15). Loss of chromosomes 13q (13q12 and 13q14-q22) and/or 16q (16q13-qter) seems to be a common genetic background in SCL (9).

Conservative surgical removal is the most indicated treatment for intraoral SCL and no recurrences are expected if the lesion is completely excised (1,9-12,14). Fregnani *et al*. (2) have shown that intraoral SCL shows a higher proliferative rate than conventional intraoral lipomas, but this does not seems to change its benign biological behavior.
